# Hospital outcomes for paediatric pneumonia and diarrhoea patients admitted in a tertiary hospital on weekdays versus weekends: a retrospective study

**DOI:** 10.1186/1471-2431-13-74

**Published:** 2013-05-11

**Authors:** David Gathara, Grace Irimu, Harrison Kihara, Christopher Maina, Dorothy Mbori-Ngacha, Julius Mwangi, Elizabeth Allen, Mike English

**Affiliations:** 1 Centre for Geographic Medicine Research – Coast, KEMRI/Wellcome Trust Research Programme, P.O. Box 230 Kilifi and P.O. Box 43640, Nairobi, Kenya; 2 Department of Paediatrics and Child Health, College of Health Sciences, University of Nairobi, P.O. Box 19676–00202, Nairobi, Kenya; 3 Kenyatta National Hospital, P.O. Box 20723–00202, Nairobi, Kenya; 4 London School of Hygiene and Tropical Medicine, Keppel Street, London, WC1E 7HT, UK; 5 Department of Paediatrics, University of Oxford, Oxford, UK

**Keywords:** Children, Pneumonia, Diarrhea, Weekend versus weekday, Quality of health care

## Abstract

**Background:**

Quality of patient care in hospitals has been shown to be inconsistent during weekends and night-time hours, and is often associated with reduced patient monitoring, poor antibiotic prescription practices and poor patient outcomes. Poorer care and outcomes are commonly attributed to decreased levels of staffing, supervision and expertise and poorer access to diagnostics. However, there are few studies examining this issue in low resource settings where mortality from common childhood illnesses is high and health care systems are weak.

**Methods:**

This study uses data from a retrospective cross-sectional study aimed at “*evaluating the uptake of best practice clinical guidelines in a tertiary hospital”* with a pre and post intervention approach that spanned the period 2005 to 2009. We evaluated a primary hypothesis that mortality for children with pneumonia and/or dehydration aged 2–59 months admitted on weekends differed from those admitted on weekdays. A secondary hypothesis that poor quality of care could be a mechanism for higher mortality was also explored. Logistic regression was used to examine the association between mortality and the independent predictors of mortality.

**Results:**

Our analysis indicates that there is no difference in mortality on weekends compared to weekdays even after adjusting for the significant predictors of mortality (OR = 1.15; 95% CI 0.90 -1.45; p = 0.27). There were similarly no significant differences between weekends and weekdays for the quality of care indicators, however, there was an overall improvement in mortality and quality of care through the period of study.

**Conclusion:**

Mortality and the quality of care does not differ by the day of admission in a Kenyan tertiary hospital, however mortality remains high suggesting that continued efforts to improve care are warranted.

## Background

### Quality of care over weekends

Quality of patient care in hospitals has been shown to deteriorate at weekends and at night [1,2] with reduced patient monitoring, poor antibiotic prescription practices and poor patient outcomes [3-5]. Poorer care and outcomes, for example higher mortality in babies delivered at weekends [6], are commonly attributed to decreased levels of staffing, supervision and expertise and poorer access to diagnostics [7]. In low-income settings acutely ill children are at highest risk of death in their first 48 hours in hospital [7]. Good quality care may therefore, be of particular importance in this period. Providing good quality care at weekends may, however, be especially difficult in lower income settings where resources are already limited.

We therefore set out to examine whether children admitted at weekends were at higher risk of death than those admitted at other times in a large Kenyan tertiary hospital, Kenyatta National Hospital (KNH). In Kenya pneumonia and dehydration secondary to diarrhoea (hereafter referred to as dehydration) are two of the most common causes of admission to health facilities and mortality in the under fives. In Kenyatta National Hospital pneumonia and dehydration contribute to 55% of the admissions and 45% of the deaths [8] in the under fives. Care for these children is defined by explicit national guidelines [9] that provide standards for assessing quality of care. We therefore also examined a secondary hypothesis, whether being admitted at the weekend with pneumonia or dehydration is associated with poorer quality of admission care, as a possible mechanism for higher mortality.

## Methods

### Study site and data collection

The data used in this analysis are from a study that took place in KNH that we have described previously [10]. In brief the hospital has four general paediatric wards each with 60 beds (240 beds in total) and approximately 14000 paediatric admissions per year aged between 0–11 years and bed occupancy is often over 100%. Most of clinical in-patient care is provided by 60–75 trainee paediatricians enrolled in a three-year postgraduate paediatric training programme and are normally supervised by 25 paediatricians. There are 126 qualified nurses on the general paediatric wards; twelve to twenty nurses per shift. At the time of the study each ward had 5–8 paediatricians, 5–8 paediatric trainees per day and 5–6 nurses per shift and this staff distribution did not differ on weekends and weekdays.

The primary study aimed at “*evaluating the uptake of best practice clinical guidelines in a tertiary hospital”* with a before and after design [10]. The interventions for the parent study aimed at improving paediatric care through provision of clinical practice guideline booklets and a 5 day training focusing on improving skills in the first 48 hours of admission and supporting guideline uptake [9]. The study spanned the period 2005 to 2009 with the periods January 2005 to December 2006 being before efforts to disseminate guidelines (pre-intervention), January 2007 to June 2008 being a phase in which guidelines and training were scaled up (implementation) and July 2008 to December 2009 a period of ongoing reinforcement activities such as audit and continuing medical education sessions (post-intervention).

#### Sampling method and process

**Figure 1:**
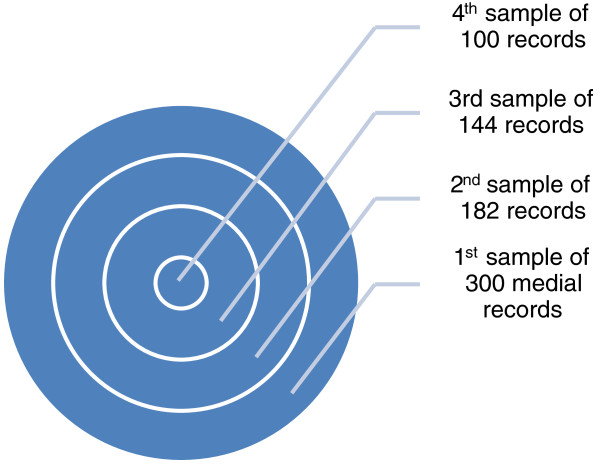
**Hierarchy of random samples.** This figure describes the multi-stage sampling process where smaller samples are sequentially nested within larger ones.

For each quarter of each year (2005–2009) a random selection of all cases with discharge diagnoses of pneumonia or dehydration was made with the aim of identifying at least 70 cases (a total of 280 cases per year) for each disease focusing on children aged 2–59 months to whom these guidelines apply, however, the selection of actual study population required that patient also have an admission diagnosis of interest. The total number of patients varied somewhat by quarter and by disease and a modified multistage random sampling technique was used to develop a hierarchy of random samples, of decreasing size, with the smaller samples nested sequentially within the bigger samples. The medical records in the smallest sample were first retrieved (identified by inpatient number) and were manually perused to eliminate those meeting exclusion criteria. If this process yielded less than 70 eligible records we retrieved the additional records that completed the next largest sample population and again manually identified those that met the inclusion criteria. If this procedure still yielded fewer than the desired sample size of 70 eligible cases, we progressed to the next largest sample population to identify further records and so on up to the largest sample in the hierarchy until sufficient eligible records were identified (Figure 1). The sampling methods and process and inclusion and exclusion criteria are described in full elsewhere [11].

Data were collected from medical records of identified cases for the period January 2005-December 2009 using a direct electronic data entry tool and detailed standard operating procedures. Data entry was evaluated daily for completeness with corrections made after re-examination of the case record. In addition, quality control was performed by the supervisor who independently re-entered a 5% sample of all abstracted records to determine agreement rates (generally higher than 95%). Any errors identified were corrected by the supervisor and any data entry clerk with an agreement rate less than 90% was retrained.

#### Sample size

The primary hypothesis explored in this analysis was whether children admitted at weekends were at higher risk of death than those admitted at other times. Assuming that approximately 30% of admissions are at weekends and based on an estimate of 7% mortality during the week (prior unpublished data from the same hospital) the available data for the analyses presented here, pooled across disease and years 2005 to 2009, would allow detection of an odds ratio for mortality associated with weekend admission of 1.6 or more with 80% power and a significance level of 5%.

#### Variable definition

A variable for the exposure, day of admission (weekend/weekday) was defined from the day and time of admission with the weekend starting Friday 6 pm (when the weekend shift starts) and ending Monday 6 am (when the weekend shift ends). While cases examined had a primary diagnosis of pneumonia or dehydration a diagnosis of both pneumonia and dehydration was observed and thus an extra category in the primary diagnosis was created for ‘both pneumonia and dehydration’ to account for situations where both of these diagnoses co-existed. Further a categorical variable for additional co-morbidities was generated and coded as either no co-morbidity or having co-morbidity. The most frequent co-morbid diagnoses being malaria, measles, rickets and anaemia. A variable for disease severity (0 = not severe/1 = severe) was generated based on the clinician’s classification of children in shock or severe dehydration for diarrhoea, those classified as very severe or severe pneumonia were identified as severe. Age was categorized into 6 month age bands. The primary outcome was death in hospital.

#### Propensity score analysis

We used propensity score analyses [12,13] to balance measurable confounders and risk factors between the exposure groups defined by day of admission to account for the non-random allocation of the exposures. Multivariable logistic regression was used to predict exposure (weekend compared with weekday) based on covariates, including gender, age, diagnosis, co-morbidities, calendar period and disease severity. Each child was then assigned an estimated propensity score, which was his/her predicted probability of admission on the weekend on the basis of his/her observed baseline characteristics. Strata were then generated based on the propensity scores with the aim of adjusting for these differences in the multivariable regression.

### Analysis

We present descriptive results as percentages and median (inter-quartile range) by day of admission, and use chi square and student t tests where appropriate for formal comparison. Univariable logistic regression was used to examine the association between the primary outcome (mortality), the main exposure of interest (day of admission) and all pre-specified covariates; gender, age, diagnosis, co-morbidities, intervention period, hospital length of stay and disease severity. In a second step, we examined for possible effect modification and confounding between mortality and weekend admission and the independent covariates using the Mantel-Haenszel method. A multivariable predictive model for mortality for children admitted on the weekend was then developed. Age and gender were included as *a priori* independent risk factors for mortality with additional variables identified in univariate analysis as associated with mortality (P < 0.05) added into the model starting with those with the strongest association. Variables were retained in the model if a likelihood ratio test (LRT, p value of <0.05) supported improved model fit. A goodness of fit test was carried out and an area under receiver operating curve (AROC) calculated to determine if the final model explained a reasonable proportion of the variability in mortality. For the univariable and multivariable analyses the odds ratios, accompanying 95% confidence intervals and Wald test p values (two-tailed) are reported.

### Further analysis

#### Quality of care

Quality of care indicators for documentation of assessment and correctness of treatment (drugs/fluids) were identified and compared against guidelines to establish if differences existed in the process of care on weekdays compared to weekends. A documentation score was calculated based on required key clinical features of either pneumonia or dehydration (for diarrhoea) as per the national clinical guidelines. For pneumonia eight key features were identified (*cough, cough duration, ability to drink/breastfeed, consciousness level, respiratory rate, cyanosis, lower chest wall in-drawing and respiratory distress*) while 5 were identified for dehydration (*sunken eyes, skin-turgor, ability to drink/breastfeed, conscious level and pulse character*). The mean and corresponding standard deviation for these scores is reported. For correctness of treatment (fluids/drugs) proportions have been reported. Since the intervention in the parent study aimed at quality of care improvement, the quality of care indicators have been reported stratified by period of intervention as described in Table 1.

**Table 1:** Definitions for quality of care indicators

	**Pneumonia**	**Dehydration**
Correctness of treatment	a) Antibiotic choice consistent with guidelines – Benzyl penicillin monotherapy for severe pneumonia in HIV negative and Benzyl penicillin and Gentamicin for very severe pneumonia and severe pneumonia in HIV positive children	a) Fluid choice consistent with guidelines – Hartman’s solution.
	b) Antibiotic dosing consistent with guidelines – 50 000 IU per kg four times a day for Benzyl penicillin and 7.5 mg twice daily for Gentamicin while allowing a 20% margin of error in the correctness of the dose prescribed.	b) Fluid volume consistent with guidelines – (+/- 20%) 80-120 mls/kg if not given fluid bolus for shock management or 56–120 mls per kg if fluid bolus for shock management is given.
		a) Fluid duration consistent with guidelines - 5–6 hours for patients ages 2–11 months and 2.5-3 hours in patients aged 12–59 months.
		b) Fluid therapy consistent with guidelines – correct choice of fluid, fluid volume and over the correct duration.

### Ethical considerations

Scientific and ethical approval for the study was obtained from the Kenyatta National Hospital Ethical Review Committee. Although the case records from which data were abstracted had names, data collected were de-identified and unique study patient identifiers created. This study was classified as an audit and therefore informed consent from the participants was not found necessary by the institutional ethics review committee. Further ethical approval was granted from the London School of Hygiene and Tropical Medicine ethical review committee to analyze these data.

## Results

**Figure 2:**
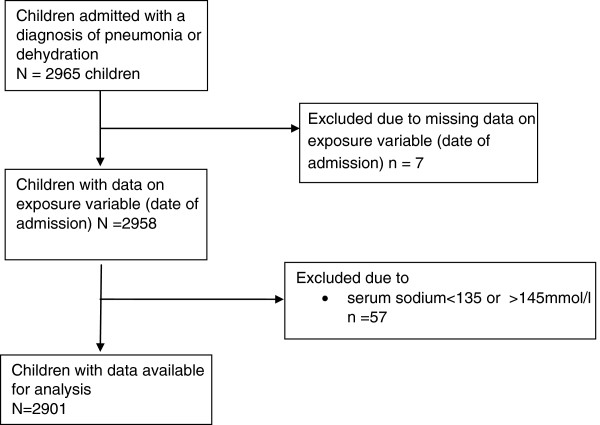
Flow diagram showing number of eligible patients and the reasons for exclusion.

A total of 2901 children with a diagnosis of pneumonia or dehydration were identified for this analysis. Of these 11% (342/2901) died during the study period, with 7% (216/2901) being on weekdays and 4% (126/2901) being on weekends. Figure 2 shows the flow diagram on recruitment of patients into the study and the reasons for exclusions.

Overall the median (IQR) age of children admitted was 8 (5–13) months with the majority 69% (1998) of children being in the age group 2–11 months. Forty nine percent (1426) had a diagnosis of pneumonia while those with dehydration and ‘pneumonia + dehydration’ were 37% (1027) and 14% (399) respectively. Further 48% (1389) had at least one co-morbidity. On average the median (IQR) length of stay was 3 (2–6) days, while the overall median time to death was 33 (13 – 87) hours for children who had the time of death and admission documented (n = 230). Of the 230 children who died and had time of death and admission documented, 60% (140) of these deaths were within the first 48 hours of admission, these and other patient characteristics are described by admission time further in Table 2. There were no statistically significant differences observed by day of admission for any of these variables. Similarly the analysis of propensity scores (predicted scores range 0.63 – 0.69) suggested only one stratum within the data demonstrating that the two groups (weekend/weekday) are comparable in terms of baseline characteristics. This suggests reported associations with admission at weekends compared with weekdays, if observed, are unlikely to be attributable to confounding or bias.

**Table 2:** Patient characteristics by day of admission

		**Weekday**	**Weekend**	**Overall**	**P value**
Mortality	n(%)*				0.298
	Alive	1689 (89)	870 (87)	2559 (88)	
	Died	216 (11)	126 (13)	342 (12)	
Gender					
	Female	859 (45)	448 (45)	1307 (45)	0.954
	Male	1046 (55)	548 (55)	1594 (55)	
Age					
	Median (IQR)	8 (5–13)	8 (5–13)	8 (5–13)	0.966
Age group					
	2 - 6 months	728 (38)	387 (39)	1115 (38)	0.425
	7 - 11 months	601 (32)	295 (30)	896 (31)	
	12 - 18 months	319 (17)	188 (19)	507 (18)	
	19 - 24 months	124 (6)	57 (6)	181 (6)	
	25 - 36 months	74 (4)	45 (4)	119 (4)	
	37 - 59 months	59 (3)	24 (2)	83 (3)	
Diagnosis					
	Dehydration	700 (37)	376 (38)	1076 (37)	0.703
	Pneumonia	947 (50)	479 (48)	1426 (49)	
	Both Dehydration and Pneumonia	258 (13)	141 (14)	399 (14)	
Hospital length of stay (LOS) days					
	Median (IQR)	3 (2 – 6)	3 (2 – 5)	3 (2 – 6)	0.845
Co-morbidities					
	No co-morbidity	982 (52)	530 (53)	1512 (52)	0.394
	With co-morbidity	923 (48)	466 (47)	1389 (48)	
Calendar period					
	2005	372 (20)	372 (20)	539 (19)	0.106
	2006	367 (19)	367 (19)	593 (20)	
	2007	369 (19)	369 (19)	555 (19)	
	2008	422 (22)	422 (22)	630 (22)	
	2009	375 (20)	375 (20)	584 (20)	
Intervention period					
	Pre-intervention	743 (39)	396 (40)	1139 (39)	0.668
	Implementation	593 (31)	294 (30)	887 (31)	
	Post-intervention	569 (30)	306 (31)	875 (30)	
Disease severity					
	Severe	754 (40)	393 (39)	1147 (40)	0.949
	Non- severe	1151 (60)	603 (61)	1754 (60)	
Time to death in hours					
	Median (IQR) n = 230	38 (13 – 86)	28 (10 – 91)	33 (13–87)	0.122

* Unless otherwise stated.

### Primary hypothesis

There was no evidence for an association between mortality and admission on weekends compared to weekdays in unadjusted analyses (OR 1.13, 95% CI 0.90 – 1.43). Several covariates including age, diagnosis, presence of additional co-morbidity and being assigned a severe classification were, however, associated with higher mortality in univariable analyses (Table 3). Further, there was a decline in the odds of death with progression of the study period. When the period of study was stratified into pre – intervention (reference), implementation and post intervention phases, there was strong evidence (p value < 0.001) for a decreased odds ratio for death of 0.61 (95% CI 0.47 – 0.80) and 0.43 (95% CI 0.32 – 0.57) for the implementation and post intervention phases respectively.

**Table 3:** Univariable analysis with mortality as the dependent variable

		**Odds ratio**	**95% CI**	**P value**
Day of admission				
	Weekday	1.00 (ref)		
	Weekend	1.13	0.90 – 1.43	0.298
Gender				
	Female	1.00 (ref)		
	Male	0.69	0.55 – 0.86	<0.001
Age group				
	2 - 6 months	1.00 (ref)		<0.001
	7 - 11 months	0.63	0.49 – 0.83	
	12 - 18 months	0.63	0.45 – 0.87	
	19 - 24 months	0.42	0.24 – 0.76	
	25 - 36 months	0.45	0.22 – 0.90	
	37 - 59 months	0.21	0.06 – 0.66	
Diagnosis				
	Dehydration	1.00 (ref)		<0.001
	Pneumonia	1.54	1.18 – 2.00	
	Both Dehydration and Pneumonia	2.32	1.66 – 3.23	
Co-morbidity				
	No co-morbidity	1.00 (ref)		
	With co-morbidity	1.65	1.31 – 2.07	<0.001
Hospital length of stay (LOS)				
	LOS in days	1.00	0.99 – 1.01	0.99
Intervention period				
	Pre-intervention	1.00 (ref)		<0.001
	Implementation	0.61	0.47 – 0.80	
	Post-intervention	0.43	0.32 – 0.57	
Disease severity				
	Non-severe	1.00 (ref)		
	Severe	1.54	1.23 – 1.93	<0.001

In the Mantel-Haenszel analysis there was weak evidence (p value 0.08) for effect modification between disease severity and day of admission but no other evidence for effect modification or confounding. Similarly in the multivariable analysis there was no evidence for an effect of weekend admission (p = 0.26) on mortality after adjusting for variables that remained significant in this model. There was however strong evidence (p <0.001) for a decrease in odds of death for each increase in age group (6 month bands) by 0.80 (95% CI 0.71 – 0.89), male gender 0.70 (95% CI 0.56 – 0.88) and an increased odds of death for diagnosis of pneumonia 1.28 (95% CI 0.97 – 1.68) and a diagnosis of ‘both pneumonia and dehydration’ 2.35 (95% CI 1.67 – 3.30) as described in Table 4. When disease severity was included as an interaction term in the multivariable model there was little evidence that the model with this term was a better fit to the data (LRT p = 0.099).

**Table 4:** Multivariable logistic regression for significant predictors of mortality

		**Odds ratio**	**95% CI**	**P value**	**LRT**
					<0.001
Day of admission					
	Weekday	1.00 (ref)			
	Weekend	1.15	0.90 – 1.46	0.258	
Age group					
	6 month bands	0.80	0.71 – 0.89	<0.001	
Gender					
	Female	1.00 (ref)			
	Male	0.70	0.56 – 0.88	0.003	
Diagnosis					
	Dehydration	1.00 (ref)		<0.001	
	Pneumonia	1.28	0.97 – 1.68		
	Both Dehydration and Pneumonia	2.35	1.67 – 3.30		
Co-morbidity					
	No co-morbidity	1.00 (ref)			
	With co-morbidity	1.83	1.43 – 2.35	<0.001	
Disease severity					
	Non-severe	1.00 (ref)			
	Severe	1.54	1.22 – 1.94	<0.001	

### Secondary hypothesis

There were no significant differences between weekends and weekdays for the quality of care indicators. Differences between weekends and weekdays in percentages in the treatment indicators were between 2% and 5% for pneumonia and 2% and 11% for diarrhoea while the mean difference for assessment was a maximum of 0.3 in both diseases. However, there was an overall improvement through the period of study for all the quality of care indicators (Table 5).

**Table 5:** Performance of quality of care indicators by weekend/weekday and intervention period

**Dehydration**	**Weekday**	**Weekend**	**Difference (95% CI)**
Adequate assessment*			
Pre-intervention; mean (sd)	1.3 (1.3)	1.5 (1.2)	+0.2 (-0.07 – 0.4)
Implementation; mean (sd)	3.1 (1.3)	2.9 (1.4)	-0.2 (- 0.5 – 0.01)
Post-intervention; mean (sd)	3.5 (1.3)	3.4 (1.2)	-0.1 (-0.4 – 0.1)
Choice of fluid consistent with guidelines			
Pre-intervention; % (n)	81 (281)	86 (163)	+5 (2 – 11)
Implementation; % (n)	79 (244)	88 (238)	+9 (4 – 16)
Post-intervention; % (n)	90 (233)	92 (132)	+2 (0.1 – 7)
Fluid volume consistent with guidelines			
Pre-intervention; % (n)	64 (225)	71 (135)	+7 (3 – 14)
Implementation; % (n)	79 (246)	75 (118)	-4 (-10 - 1)
Post-intervention; % (n)	66 (173)	77 (111)	+11 (6 – 19)
Fluid therapy consistent with guidelines			
Pre-intervention; % (n)	39 (137)	45 (85)	+6 (2 – 13)
Implementation; % (n)	52 (158)	57 (87)	+3 (0.1 -8)
Post-intervention; % (n)	61 (159)	66 (95)	+5 (2 – 11)
Pneumonia			
Adequate assessment*			
Pre-intervention; mean (sd)	3.9 (1.1)	4.1 (1.1)	+0.2 (0.02 – 0.4)
Implementation; mean (sd)	5.0 (1.3)	5.3 (1.1)	+0.3 (0.05 – 0.5)
Post-intervention; mean (sd)	5.5 (1.2)	5.5 (1.1)	0.003 (-0.2 – 0.2)
Antibiotic choice consistent with guidelines			
Pre-intervention; % (n)	14 (51)	11 (21)	-3 (-8 – 0.1)
Implementation; % (n)	35 (96)	30 (40)	-5 (-11 - 2)
Post-intervention; % (n)	46 (139)	48 (76)	+2 (0.1 – 7)
Dosage consistent with guidelines			
Pre-intervention; % (n)	60 (220)	64 (119)	+4 (1–10)
Implementation; % (n)	92 (230)	93 (107)	+1 (0 – 5)
Post-intervention; % (n)	95 (267)	91 (138)	-4 (-10 - 1)

*Adequate assessment difference is based on the means while that for correct treatment is proportions.

## Discussion

This study aimed at evaluating whether mortality differed by the day of admission for children with diagnoses of pneumonia or diarrhoea. We hypothesised that due to the limited resources and staff available over weekends, weekend admissions could be associated with greater hospital mortality. Further, we hypothesised that weekend admission might be associated with poor quality of care, a possible mechanism for higher mortality. Our findings from both crude and adjusted analyses did not support these hypotheses. However, significant independent predictors of mortality were identified and included age, gender, presence of co-morbidity, assigned disease severity and diagnosis (with pneumonia and ‘both pneumonia and dehydration’ increasing the risk of death compared to dehydration alone) consistent with findings from other studies [7,14].

The apparent absence of an effect might result from misclassification of exposure status since we were able to classify children as admitted at the weekend primarily by day of admission. However similar challenges in provision of care (our real exposure of interest) may occur during out-of-office hours (night-time hours) on weekdays. Unfortunately poor quality data available on time of admission prevented us from identifying this potential risk group and thus they were considered part of the weekday admissions. As well as methodological explanations, the absence of effect might reflect the continuous availability of paediatric residents consistent with the teaching status of the hospital, consistent with findings from other studies on teaching hospitals [15]. Finally our results may suggest that basic forms of care are similarly provided at all times, as evidenced by the absence of difference in quality measures, and that reducing relatively high mortality rates may require additional resources or interventions independent of the day of admission.

Overall there was a decline in mortality and an improvement in the quality of care indicators through the three intervention periods which is consistent with the report from the parent study on the evaluation of the intervention [10]. The parent study’s before and after design means however, that inferring a causal link between intervention and improved outcomes is undermined by potential bias and confounding. In contrast comparing the effect of admission day on mortality and quality of care over the whole course of the study should allow us to detect an underlying, time-invariant effect if one exists at the magnitude hypothesised.

### Strengths and limitations

The results reported are from a large sample size collected over a period of 5 years with cases randomly selected and abstracted by trained data abstractors and is among the first such studies in sub-Saharan Africa. Some of the draw backs of similar previous studies have been the challenge of adjusting for disease severity. We attempted to take this into account, although partially, as we had to rely on clinicians assigning patients a ‘severe’ category, something that is hard to standardise, and their documentation of co-morbidity. We examined a potential mechanism for variations in mortality by exploring possible differences in quality of care. For both diseases analyses were consistent in showing no evidence of a weekend effect on our indicators of quality. Further the propensity score findings suggest that our results are not confounded by patient and other characteristics at admission.

However these results should be interpreted in the light of the following limitations. Firstly, pre-hospital information such as prior treatment/interventions administered and whether children had been referred from lower level facilities were not available and these factors may affect mortality. Secondly, this is a retrospective study with data abstracted from routine case records. Poor documentation of care, often seen in hospitals in developing countries [16-18], may result in misclassification of exposures and undermine the results. Thirdly, these data are only from the main teaching and referral hospital in Kenya meaning that these results may not be generalizable to all Kenyan hospitals but may be extrapolated to other teaching and referral hospitals in the region. Despite these limitations and the absence of an obvious effect of admission day on measured outcomes, the data do suggest there is a general need to continue to improve quality of care over weekends and weekdays in a tertiary teaching hospital.

## Conclusion

Mortality and the quality of care for children with pneumonia or dehydration in a large Kenyan teaching hospital does not differ by the day of admission. However mortality remains high suggesting that continued efforts to improve care are warranted.

## Competing interests

The authors declare that they have no competing interests.

## Authors’ contributions

The idea for the primary study and its design were conceived by Grace Irimu (GI), with advice from Mike English (ME). ME obtained the funding for this project. GI and ME provided and coordinated training and guideline dissemination; David Gathara (DG) and GI were responsible for data collection. DG, EA, and ME were responsible for data analyses and DG, EA, and ME prepared the initial draft manuscript. All authors reviewed the draft manuscript and provided input to and approval for the final version of the report.

## Pre-publication history

The pre-publication history for this paper can be accessed here:

http://www.biomedcentral.com/1471-2431/13/74/prepub
